# Temporal changes in cortical microporosity during estrogen deficiency associated with perilacunar resorption and osteocyte apoptosis: A pilot study

**DOI:** 10.1016/j.bonr.2022.101590

**Published:** 2022-05-17

**Authors:** H. Allison, L.M. O'Sullivan, L.M. McNamara

**Affiliations:** Mechanobiology and Medical Devices Research Group (MMDRG), Centre for Biomechanics Research (BioMEC), Biomedical Engineering, College of Science and Engineering, National University of Ireland Galway, Ireland

**Keywords:** MMP, Matrix metalloproteases, PLR, Perilacunar resorption, Micro-CT, Micro computed tomography, OVX, Ovariectomized, BSEM, Backscattered scanning electron microscopy, BMDD, Bone mineral density distribution, Lc, Lacunar, Tb, Trabecular, Th, Thickness, Sp, spacing, BV, Bone volume, TV, Total volume, V Ca, Vascular canal, Dm, Diameter, TRAP, Tartrate-resistant acid phosphatase, με, Microstrain (ε ×10^−6^), Microporosity, Estrogen deficiency, micro-CT, Lacunar, Vascular canals, BSEM

## Abstract

Osteocytes can actively regulate bone microporosity, through either perilacunar resorption or micropetrosis following apoptosis. Osteocyte apoptosis is more prevalent in estrogen deficiency and changes in the lacunar-canalicular network of osteocytes have been reported. Temporal changes in bone mineralisation and osteocytes cellular strains occur, which might be associated with osteocyte-driven microporosity changes, although time dependant changes in bone microporosity are not yet fully understood. In this pilot study we conducted micro-CT analysis, backscatter electron imaging and histological analysis of femoral cortical bone form an ovariectomized rat model of osteoporosis to investigate whether estrogen deficiency causes temporal changes in lacunar and vascular porosity. We also assessed MMP14 expression, lacunar occupancy and mineral infilling, as indicators of perilacunar resorption and micropetrosis. We report temporal changes in cortical microporosity in estrogen deficiency. Specifically, canalicular and vascular porosity initially increased (4 weeks post-OVX), coinciding with the period of rapid bone loss, whereas in the longer term (14 weeks post-OVX) lacunar and canalicular diameter decreased. Interestingly, these changes coincided with an increased prevalence of empty lacunae and osteocyte lacunae were observed to be more circular with a mineralised border around the lacunar space. In addition we report an increase in MMP14+ osteocytes, which also suggests active matrix degradation by these cells. Together these results provide an insight into the temporal changes in cortical microporosity during estrogen deficiency and suggest the likelihood of occurrence of both perilacunar resorption and osteocyte apoptosis leading to micropetrosis. We propose that microporosity changes arise due to processes driven by distinct populations of osteocytes, which are either actively resorbing their matrix or have undergone apoptosis and are infilling lacunae by micropetrosis.

## Highlights

• Cortical canaliculi and vascular porosity increased during short term estrogen deficiency, coinciding with the period of rapid bone resorption
• Lacunar-canalicular porosity reduced in long term estrogen deficiency
• In the longer term empty lacunae were also observed, which were circular with mineralised borders, and there was an increase in MMP14+ osteocytes

## 1 Introduction

Microporosity in cortical bone is dictated by vascular pores, which surround blood vessels and nerves, and lacunar-canalicular channels, within which osteocytes and their dendritic processes reside. The lacunar-canalicular network and vascular porosity are interconnected, and interstitial fluid flow occurs and enables transportation of nutrients and signalling molecules and removal of waste products. Vascular canals act as low-pressure reservoirs for fluid drainage from osteocyte lacunae and canaliculi (Cowin and Cardoso, 2015; Wang et al., 1999; Goulet et al., 2008). Fluid flow within the lacunar-canalicular network induces shear stress on osteocytes and their protein-based tethers to the extracellular matrix, which serve to transmit mechanical stimuli to the osteocyte cell membrane and processes that can induce osteocyte mechanotransduction (Han et al., 2004; Wang et al., 2007; McNamara et al., 2009).

Microporosity changes arise due to intracortical remodelling, either by osteoclast resorption or by perilacunar remodelling by osteocytes (Langdahl et al., 2016; Zebaze et al., 2014; Lloyd et al., 2014). Osteocytes can resorb their surrounding perilacunar and extracellular matrix and mineral by releasing matrix metalloproteases, ATPase proton pumps and enzymes, in particular Cathepsin K (Kaya et al., 2017; Qing et al., 2012; Yee et al., 2019; Kogawa et al., 2013). Mineral infilling of the empty lacunae that remain after osteocyte apoptosis can also arise and is known as micropetrosis (Frost, 1960; Busse et al., 2010; Tomkinson et al., 1998). During ageing osteocyte lacunae become more spherical and osteocyte lacunar volume, density and canalicular area decrease (Ashique et al., 2017; Vashishth et al., 2000; Busse et al., 2010; Hemmatian et al., 2018; Tiede-Lewis et al., 2017; Kobayashi et al., 2015). The expansion of existing vascular canals and merging of large pores also occurs with age in humans, particularly at the endosteal surface (Cooper et al., 2007).

Studies have been conducted to determine changes in osteocyte density and lacunar occupancy (an indicator of osteocyte death) in trabecular bone from human transiliac bone biopsies of osteoporotic and control subjects by histomorphometry (Mullender et al., 1996; Mullender et al., 2005). Using these approaches it first reported that lacunar and osteocyte number per bone area were significantly reduced in osteoporotic patients (male and female pooled) relative to control (Mullender et al., 1996). In a later study of bone tissue from osteoporotic women, significantly lower osteocyte numbers and lacunae per bone area were reported, when compared to bone tissue from healthy women (Mullender et al., 1996). In a separate study osteocyte lacunae shape and size were assessed from femoral trabecular bone of women with and without osteoporotic fracture by confocal microscopy of a site of high fracture risk, and no significant changes in lacunar area were reported (McCreadie et al., 2004). For each of these studies, only one biopsy was taken for analysis, and the studies were confounded by variability in patient age and location chosen for analysis and, most importantly, the extent of disease in these patients was unknown.

The osteocyte and lacunar-canalicular network was analysed in bone tissue from animal models of osteoporosis. Female Sprague-Dawley OVX rats were sacrificed after 6 months of ovariectomy induced bone loss, and at this single time-point lacunar porosity analysis was conducted (Tommasini et al., 2012). They reported that lacunar volume was significantly lower and lacuna number density was significantly higher at the endosteal surface of OVX cortical femoral diaphysis compared to that of age-matched controls (Tommasini et al., 2012). In a separate study lacunar-canalicular and vascular porosity were analysed in the cortical and cancellous metaphysis bone tissue at a single time-point (6 weeks) after loss of ovarian function in female Sprague Dawley rats (Sharma et al., 2012; Sharma et al., 2018). It was reported that canalicular and vascular porosity were increased in OVX rats, but there was no significant difference in osteocyte lacunar density, lacunar size or the number of canaliculi per lacuna (Sharma et al., 2012; Sharma et al., 2018). Osteocyte densities and osteocyte size were measured from backscattered electron scanning electron microscopy images of cortical bone at 4 and 8 weeks post-ovariectomy in Sprague Dawley rats, that were ovariectomized at 12-weeks of age, and no differences were reported (Shah et al., 2018). Variations in experimental methods, location of bone used for analysis or animal age at time of ovariectomy might explain discrepancies between previous studies. Alternatively, the osteocyte lacunar density and lacunar canalicular network might vary depending on the extent of the disease progression. However, time dependant changes in lacunar-canalicular of vascular porosity are not fully understood.

There is emerging evidence that osteoporosis is not simply associated with bone loss, but that time-dependant changes in bone tissue structure and composition arise. In vivo micro-CT studies have revealed that although rapid bone loss occurs within the first month of estrogen deficiency in the proximal tibia of OVX rats (Boyd et al., 2006; Perilli et al., 2010; Brouwers et al., 2008; Waarsing et al., 2006; O'Sullivan et al., 2020), secondary changes in bone mineralisation and trabecular thickening occur in longer term estrogen deficiency (Perilli et al., 2010; Brouwers et al., 2008; Waarsing et al., 2006; O'Sullivan et al., 2020). In particular it was shown that changes in bone mineralisation and osteocytes cellular strains occur, whereby the osteocyte mechanical environment was altered in ovariectomized rats during the early stages of estrogen deficiency (5 weeks post-OVX), but were restored in the longer term (Verbruggen et al., 2015). Osteocyte apoptosis has also been reported to increase in osteoporotic bone (Tomkinson et al., 1998; Almeida et al., 2007; van Essen et al., 2007; Emerton et al., 2010), and might lead to micropetrosis driven mineralisation of the osteocyte microenvironment. Such osteocyte-driven changes in microporosity might explain temporal and heterogeneous mineralisation in estrogen deficiency (O'Sullivan et al., 2020). However, whether temporal changes in lacunar and vascular porosity arise in estrogen deficiency has never been investigated.

In this study we test the hypothesis that estrogen deficiency causes temporal changes to both the vascular porosity and lacunar-canalicular network in cortical bone, which are associated with perilacunar resorption, osteocyte apoptosis and micropetrosis. The objectives of this study are to (1) characterise trabecular microarchitecture and mineral distribution within trabecular and cortical bone of the distal femur, (2) characterise temporal changes in lacunar and vascular porosity of femoral cortical bone and (3) assess MMP14 expression, lacunar occupancy and mineral infilling, as indicators of perilacunar resorption and micropetrosis associated with osteocyte apoptosis.

## 2 Materials and methods

### 2.1 Animal model

All animal procedures were carried out under license from the Animal Care and Research Ethics Committee (ACREC) of the National University of Ireland, Galway and the Health Products Regulatory Authority (HPRA), the national authority for scientific animal protection in Ireland. Female retired breeder Wistar rats (6 month old, Charles River, Ireland) were randomly assigned to groups for either (a) bilateral ovariectomy (OVX, *n* = 8) or (b) a sham operation (SHAM, n = 8) (Body weight, OVX: 420 ± 66 g, SHAM: 438 ± 29 g, mean ± standard deviation). Successful ovariectomy was confirmed in necropsy by the absence of ovaries and determining atrophy of the uterine horns. To avoid an increase in appetite induced by the removal of the ovaries, food was restricted (22 g/animal/day) for animals below a weight of 500 g under the advice of the Animal Welfare Officer, and for animals above 500 g the diet was adapted to not feed animals under 85–90% of free feeding (5 g/100 g). Water was provided and taken by animals at libitum. These OVX and SHAM animals were sacrificed by CO_2_ asphyxiation at (1) 4 weeks post-ovariectomy (short term estrogen deficiency) (OVX: *n* = 4, SHAM: n = 4) or (2) 14 weeks post-ovariectomy (long term estrogen deficiency) (OVX: n = 4, SHAM: n = 4). The right femurs were harvested, and fixed in 10% formalin and used for micro-CT analysis, histology and backscatter electron imaging.

### 2.2 Micro-Ct scanning

The right distal femur (OVX, week 4: n = 4, week 14: n = 4 and SHAM, week 4: n = 4, week 14: n = 4) were excised and scanned ex vivo at an isotropic resolution of 2 μm by micro-CT (μCT 100, Scanco Medical AG, Basserdorf Switzerland). The following scan setting were used: x-ray tube potential of 70 kVp, current 57 μA, integration time 1500 ms, 1500 projections per 180° and a 0.5 mm thick aluminium filter was used to reduce beam hardening artefacts.

**Fig. 1:**
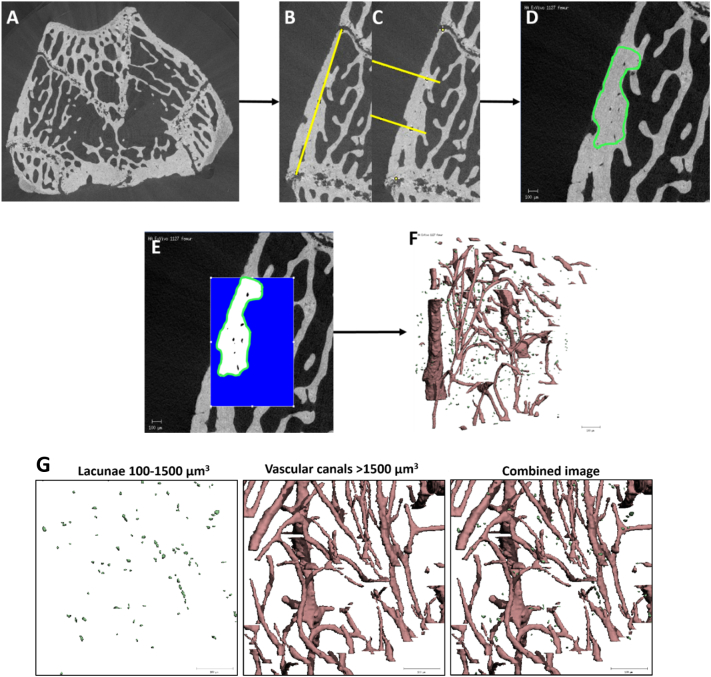
Micro-CT analysis from DICOMS to 3D reconstructions of cortical micro-architecture and μCT-derived cortical bone microporosity. (A) A series of DICOMS are taken from each scan, (B) Anterior and posterior growth plate on the medial side of the cortical bone is defined (yellow line) in ImageJ, (C) ImageJ script defines middle third of the cortical bone (two yellow lines), (D) middle third outlined in ImageJ is then used as a template for the segmentation process, (E) Bone is segmented from other components using a global threshold of 780 mg/HA ccm, (F) Intracortical porosity is segmented by inverting the image, (G) 3D microporosity reconstruction in distal femur of a week 14 SHAM animal. Volume filtering removed noise (<100 μm^3^) and pores were classified as lacunae (100–1500 μm^3^) and vascular canals (>1500 μm^3^). Scale bar = 100 μm.

To ensure consistent analysis and comparison between both groups, a region of interest on the medial side of the bone was selected for analysis (Fig. 1). The medial region was chosen because in healthy animals this region is dense in trabecular bone and undergoes high bone loss in osteoporosis, and also to avoid ligament attachment sites on the lateral side of the bone. The anterior and posterior growth plates were used as reference points to define the region, which comprised of a 1 mm section of metaphyseal trabecular bone, 0.5 mm away from the growth plate in the distal femur bone. To prevent operator bias and ensure consistent comparison between groups an ImageJ script was developed to define the medial and lateral side of the trochlear groove and the anterior and posterior growth plate on the medial side of the distal femur in a series of DICOMs. The regions for analysis were defined using a semi-automated segmentation process whereby the user defined borders around the trabecular network or the medial cortical bone region from the image series, which were then extrapolated across all intervening images using a dynamic interpolation algorithm in the Scanco segmentation software (Fig. 1). After the region was defined the data was segmented and processed further for analysis. For this purpose bone was separated from the other components present in the micro-CT scans (i.e. vascular canals, lacunae and air) using a global threshold of 780 mg/HA ccm. A Gaussian filter was applied (σ = 0.8; support = 1) to reduce noise and ring artefacts (Bouxsein et al., 2010). Trabecular bone analysis was conducted on the anterior medial half of the trabecular bone and cortical bone analysis was conducted on the middle third cortical bone region.

### 2.3 Morphometric analysis

A series of micro architectural parameters were quantified from the three-dimensional reconstruction of the trabecular volume of interest, using the evaluation scripts in the Scanco IPL. Bone volume fraction (BV/TV) and 3D trabecular thickness (Tb.Th) were quantified to describe the bone mass and its distribution. Trabecular number (Tb.N) and trabecular separation (Tb.Sp) were analysed to complete a detailed description of the trabecular structure in the distal femur of the OVX and SHAM groups.

### 2.4 Microporosity analysis

A series of microporosity parameters were quantified from the three-dimensional reconstruction of the cortical region of interest, using evaluation scripts in the Scanco Image Processing language (IPL). Intracortical porosity comprising of lacunae and vascular canals were segmented by inverting the image. Objects with a volume less than 100 μm^3^ were considered to be noise, elements with a volume in the range between 100 and 1500 μm^3^ were assumed to be osteocyte lacunae, whereas objects with a volume greater than 1500 μm^3^ were considered to be vascular canals, see Fig. 1(G). These volume limits were used in previous nano-CT and synchrotron-based studies (Carriero et al., 2014; Javaheri et al., 2015; Mosey et al., 2017) and were based on confocal microscopy measurements that indicated that osteocyte size was between 28 and 1713 μm^3^, with only 0.32% of the lacunae analysed under 100 μm^3^ and 99% of all lacunar volumes were within 100–600 μm^3^ (McCreadie et al., 2004; Tommasini et al., 2012).

These parameters allow for detailed comparison between cortical microporosity of the estrogen deficient (OVX) and healthy (SHAM) animals at week 4 and 14. Lacunar porosity (Lacunar volume/total volume) and lacunar density (N. Lacunae/total volume) were quantified to characterise lacunae present in the cortical bone. Vascular canal diameter (μm), vascular canal porosity (Canal volume/total volume) and vascular canal density (N. Canals/total volume) were quantified to characterise vasculature in the bone.

### 2.5 Bone mineral density distribution analysis

Bone mineral density distribution analysis allows for assessment of the amount of bone volume at various stages of mineralisation and has been used to assess the changes in bone composition during estrogen deficiency (O'Sullivan et al., 2020). To assess changes in cortical and trabecular bone mineralisation the raw micro-CT normalised tissue mineral density (mg HA/ccm) data were quantitatively processed to create bone mineral density distribution (BMDD) measurements for each of the data sets. Grey level histograms were produced from the data to analyse the frequency of occurrence of voxels at a certain grey level or tissue mineral density (mg HA/ccm). To prevent partial volume effects from causing an underestimation of the cortical bone mineral density, the surface voxels (two layers) from each cortical section and from each trabecula are excluded from these histograms. A custom Python script was used to characterise specific characteristics of each distribution: weighted mean tissue mineral density (mean mineral density, mgHA/ccm) and the full width at half maximum of the mineral distribution curve (mineral heterogeneity, mg HA/ccm), following similar approaches developed in our laboratory (O'Sullivan et al., 2020).

### 2.6 Histological sample processing

Transverse sections of metaphyseal bone (1.5 mm) were cut from the fixed distal femurs, using a diamond blade low speed saw (Isomet™, Buehler, IL, USA). These sections were incubated in 10% di- and tetra-sodium Ethylenediaminetetraacetic acid (EDTA) for 14 days until fully decalcified. The decalcification endpoint was determined using an oxalate test and a physical probing test. After decalcification, samples were rinsed in PBS overnight. Afterwards samples were processed using a tissue processor (Leica ASP300) following a routine protocol (formalin, ascending grades of ethanol, xylenes and paraffin immersion) and then embedded in paraffin (Leica EG1150H). Paraffin sections (8 μm thick) were generated using a microtome (Leica RM2235). Slices were collected on SuperFrost® Plus slides (Menzel Glaser), and stored at room temperature, until staining.

### 2.7 MMP14 staining

Matrix metalloproteinases (MMPs) play an essential role in degrading matrix during peri-lacunar remodelling, and MMP14 (also known as MT1-MMP) has been identified to play a role in osteocyte canalicular network development and maintenance (Holmbeck et al., 2005). MMP14 staining has been used previously to identify osteocytes that were actively involved in peri-lacunar remodelling (Qing et al., 2012; Kogawa et al., 2013; Dole et al., 2017; Yee et al., 2019; Delgado-Calle et al., 2018). To assess changes in perilacunar remodelling, MMP14 staining of osteocytes was conducted in the metaphyseal cortical bone sections from the medial side of the fixed distal femora. Slides were deparaffinised in xylene, and rehydrated in descending grades of ethanol (100, 90, 70, 50 and 30). Antigen retrieval was performed using proteinase K in TE (Tris-EDTA) buffer. Slides were incubated in the retrieval solution for 20 min at 37 °C in a humidified chamber. Slides were then cooled down for 10 min before being rinsed twice with PBS-Tween 0.5% *v*/v (Sigma-Aldrich). Blocking solution was made using normal goat serum (NGS) 10% *w*/*v* and bovine serum albumin (BSA) 1% w/v in PBS. Slides were blocked for 1 h at room temperature. After blocking, slides were incubated with the primary antibody anti-MMP14 (1:100; Abcam, ab38971) overnight at 4 °C. Slides were then rinsed with TBS (Tris-buffered saline) 0.025% TritonX-100, and endogenous peroxide activity was quenched using 0.3% H_2_O_2_ in TBS for 15 min. Slides were then washed three times with 1% BSA solution, before being incubated with the secondary antibody (Goat Anti-Rabbit IgG H&L (HRP); Abcam, ab6721) for 1 h at room temperature. Slides were washed 3 times with 1% BSA solution before being incubated with fresh DAB solution (Vector Laboratories) and incubated for 5 min. Slides were washed with deionised water and dehydrated using ascending ethanol series, mounting was performed using Organo/Limonene Mount™ (Sigma-Aldrich). To evaluate specificity, controls containing antibody diluent instead of primary antibodies were used with the secondary antibody. Slides were imaged on a light microscope (Olympus BX43, Olympus, Tokyo, Japan). The prevalence of positively stained osteocytes, normalised to total bone area was assessed using ImageJ, from multiple histological sections of metaphyseal cortical bone from the medial side of the distal femora, with *n* = 4 femurs from each group.

### 2.8 Haematoxylin and Eosin staining

Slides were deparaffinised using xylene, then rehydrated using descending grades of ethanol (100, 90, 70%) and then rinsed in tap water. Slides were subsequently immersed in Mayer's Haematoxylin (Sigma-Aldrich), before being dipped in deionised water 3 times and immersed in tap water to remove excess stain. Slides were dipped three times in HCL/ethanol solution (1% HCL in 70% ethanol) and immersed in tap water before being stained with Eosin (Thermo scientific) for 3 min. Slides were then washed again with several deionised water rinses before being dehydrated with ascending ethanol series, mounting was performed using Organo/Limonene Mount™ (Sigma-Aldrich). Slides were imaged on a light microscope (Olympus BX43, Olympus, Tokyo, Japan). The percentage of empty lacunae was analysed on ImageJ from multiple histological sections of metaphyseal cortical bone from the medial side of the distal femora, with *n* = 4 femurs from each group.

### 2.9 TRAP staining

Slides were deparaffinised using xylene, then rehydrated using descending grades of ethanol and then rinsed in tap water. Slides were then incubated in pre-warmed TRAP staining solution (containing Sodium acetate anhydrous, L-(+) Tartaric acid, Glacial Acetic acid, Napthol AS-MX Phosphate, Ethylene Glycol Monoethyl Ether and Fast Red Violet LB salt) for 30 min at 37 °C. Slides were then rinsed with deionised water before being counter-stained with 0.02% Fast Green Solution for 30 s. Slides were rinsed and then dehydrated with graded ethanol, after which mounting was performed using Organo/Limonene Mount™ (Sigma-Aldrich). Slides were imaged on a light microscope (Olympus BX43, Olympus, Tokyo, Japan). The number of TRAP+ cells/mm was quantified on ImageJ from multiple histological sections of metaphyseal cortical bone from the medial side of the distal femora, with n = 4 femurs from each group. Staining was performed on RAW264.7 cells as a positive control.

### 2.10 Backscatter scanning electron microscopy

Femoral distal metaphyseal bone (n = 4 rats/per group) were cut transversely using a diamond blade low speed saw (Isomet™, Buehler, IL, USA) to prepare medial bone samples for BSEM analysis. Bone marrow was removed using PBS, via syringe and needle, to ensure epoxy infiltration. Bone sections were then dehydrated using ascending grades of ethanol and then incubated in hexamethyldisilazane (HMDS) (Sigma-Aldrich) for 30 min and left to air dry. The samples were then suspended in moulds (Buehler, IL, USA) and embedded in epoxy resin (EpoThin 2, Buehler, IL, USA) and placed in a vacuum for 2 min to facilitate epoxy infiltration into the marrow space, samples were then allowed to harden at room temperature for 3 days. Embedded samples were then cut at either end to create a parallel surface using a diamond blade low speed saw and placed in an ultrasonic bath (VWR, Dublin, Ireland) at room temperature for 5 min. Samples were polished using the MetaServ 250 polishing system (Buehler, IL, USA), with diamond powder suspensions of decreasing particle size (9, 3, 1 μm) ending with a final size of 0.05 μm, samples were sonicated in between each polishing step for 5 min. Backscattered electron imaging was performed on a Hitachi S-2600 system with a 4-segment solid state BSE detector (Hitachi, Tokyo, Japan). Images were taken at 20 kV accelerating voltage, and a beam current of 80 pA under a vacuum pressure of 50 Pa. Then ≥15 lacunae per specimen were randomly selected in a similar area to the micro-CT work (i.e. medial cortical bone, middle third of the bone). Images were captured at 3000× magnification to measure the length of minor and major lacunae diameters. Images were also taken at 100× to assess cortical thickness, 200× to assess percentage area taken up by lacunae and vascular pores, and 6000× to measure canalicular diameter. Hitachi built-in software was used to measure the length of the minor and major lacunae diameters, canalicular diameter and cortical thickness. Image J was used to quantify percentage area taken up by lacunae or vascular pores, in which images were colour thresholded and percentage area was quantified.

### 2.11 Statistical analysis

Data is presented as box plots and whiskers is as follows: whiskers extend between min and max values of data set, the box extends from 25th to 75th percentiles, the horizontal line across the box is the median value and the black dot represents the mean of the data. Statistical analysis was performed by unpaired two-tailed Student's *t*-test. A value of *p* < 0.05 was regarded as statistically significant. Micro-CT, backscatter electron imaging and histological analysis was performed on 4 rats per group, which was determined based on a power analysis to detect a 45% decrease in BV/TV for the OVX group compared to SHAM at 4 weeks from, giving a power of 0.90. For backscatter electron imaging and immune/histological analysis multiple sections and osteocytes lacunae were imaged per animal. For statistical analysis measurements were averaged per animal and *n* = 4 for all the statistical analyses.

## 3 Results

### 3.1 Trabecular bone loss, mineralisation and osteoclastogenesis in the distal femur of estrogen deficient rats

**Fig. 2:**
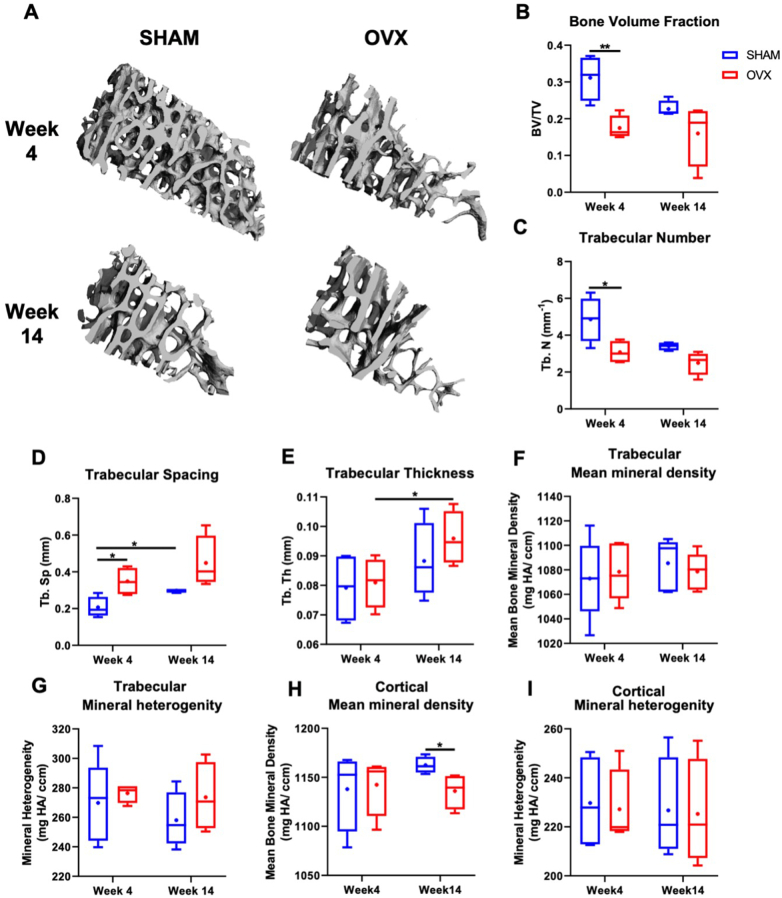
(A) 3D micro-CT images of an area of metaphyseal trabecular bone in the distal femur of one representative OVX animal after short (4 weeks) and long term (14 weeks) estrogen deficiency and the SHAM operated counterpart. Quantitative analysis of micro-CT data from week 4 and week 14 SHAM and OVX animals for (B) Bone volume fraction (BV/TV), (C) Trabecular number (mm^−1^), (D) Trabecular spacing (mm), (E) Trabecular thickness (mm), (F) trabecular mean mineral density (mg HA/ccm), (G) trabecular mineral heterogeneity (mg HA/ccm), (H) cortical mean mineral density (mg HA/ccm), (I) cortical mineral heterogeneity (mg HA/ccm). Boxplots and whiskers display the following: whiskers extend between minimum and maximum values of the dataset, the box extends from 25th to 75th percentiles, horizontal line across the box is the median value and the black dot represents the mean of the data. *n* = 4 per group. * = *p* < 0.05 and ** = *p* < 0.01.

We analysed the changes in trabecular morphometry and bone mineralisation that occur due to estrogen deficiency by performing micro-CT analysis on the distal femur of short and long term estrogen deficient animals (Fig. 2A). Bone volume fraction was significantly reduced (*p* < 0.01) (Fig. 2B), and there was a significant reduction in trabecular number and an increase in trabecular spacing in week 4 OVX animals compared to week 4 SHAM animals (*p* < 0.05) (Fig. 2C, D). No changes in trabecular thickness were observed in short term estrogen deficiency. However, a significant increase in trabecular thickness was observed in week 14 OVX animals compared to week 4 OVX animals (p < 0.05) (Fig. 2E). There was no statistically significant difference in the trabecular morphometry parameters between groups by week 14.

In addition to changes in the microstructural properties of cortical bone during early stage estrogen deficiency, region specific changes in mineral content have also been observed (Sharma et al., 2018). There was a decrease in cortical mean mineral density in week 14 OVX animals compared to week 14 SHAM animals (p < 0.05) (Fig. 2H), but no other significant differences in mean mineral density or mineral heterogeneity were reported for cortical or trabecular bone from the distal femur of OVX and SHAM in short or long term estrogen deficiency (Fig. 2F, G, H, I).

**Fig. 3:**
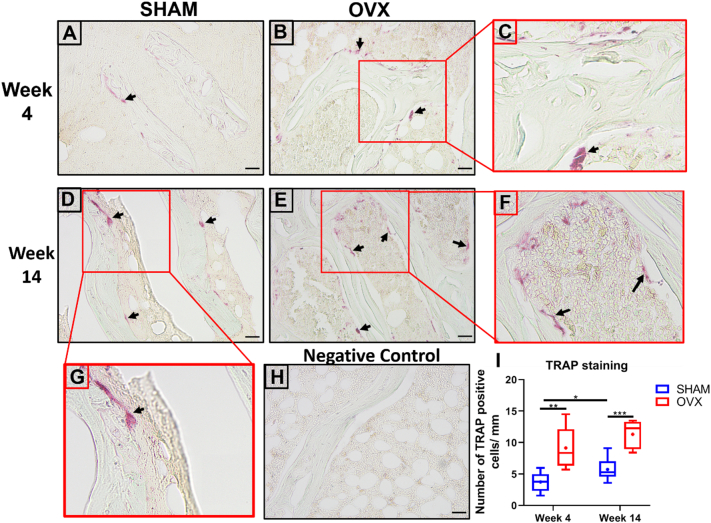
OVX rats display increased TRAP+ staining compared to SHAM animals. Representative image showing TRAP staining and fast green counterstain of cortical bone from (A) week 4 SHAM animals, (B) week 4 OVX animals, (C) magnified image (red box) of week 4 OVX animals, (D) week 14 SHAM animals, (E) week 14 OVX animals, (F) magnified image (red box) of week 14 OVX animals (G) magnified image (red box) of week 14 SHAM animals, (H) Negative control = counterstain only and (I) ImageJ analysis was performed to quantify number of TRAP+ cells per mm. n = 4 rats/group. Scale bar = 20 μm. * = p < 0.05, ** = p < 0.01 and *** = *p* < 0.001. (For interpretation of the references to colour in this figure legend, the reader is referred to the web version of this article.)

TRAP staining was performed to assess temporal changes in osteoclast number in OVX rats compared to SHAM animals. As expected, increased TRAP staining was seen in week 4 OVX animals compared to controls (week 4 SHAM) (p < 0.01) (Fig. 3A, B, C, I). There was an increase in TRAP+ cells in week 14 SHAM animals compared to week 4 SHAM animals (*p* < 0.05) (Fig. 3A, D, G, I). Additionally, increased TRAP staining was observed in long term estrogen deficient animals (week 14 OVX) compared to week 14 SHAM animals (Fig. 3D, E, F, G).

**Fig. 4:**
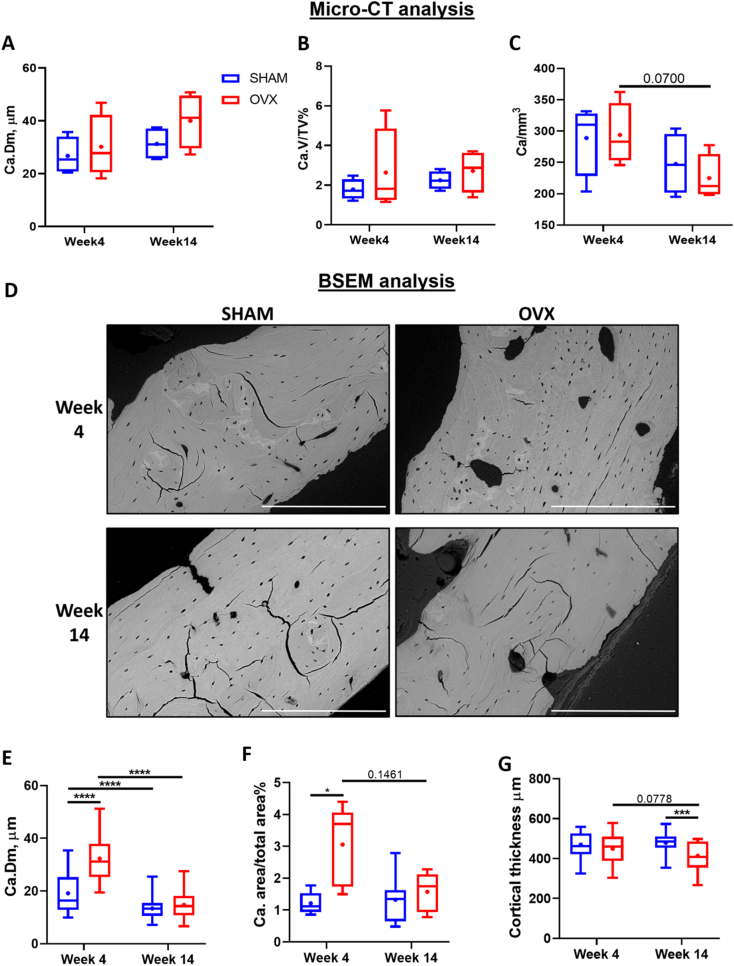
Micro-CT vascular microporosity measurements for cortical bone from the medial region of the distal femur metaphysis for SHAM and OVX groups after short (4 weeks) and longer term (14 weeks) of estrogen deficiency. (A) Vascular diameter, Ca.Dm (μm), (B) Vascular porosity, Ca.V/TV (%) and (C) Vascular density, Ca/mm^3^. (D) Representative BSEM images of cortical bone from the medial region of the distal femur metaphysis for SHAM and OVX groups at early (week 4) and late (week 14) estrogen deficiency, which were analysed to quantify (E) average vascular canal diameter (μm), (F) percentage area taken up by vascular canals in a given area (%) and (G) average cortical thickness (μm). Lc = lacunar, Ca = vascular canal, TV = total volume, Dm = diameter. n = 4 animals per group. Scale bar = 200 μm. * = p < 0.05, *** = p < 0.001 and **** = *p* < 0.0001.

By BSEM analysis, we report that there were no significant differences in cortical thickness between SHAM and OVX animals at week 4. However, a significant decrease in cortical thickness was observed in week 14 OVX animals compared to SHAM week 14 animals (*p* < 0.001) (Fig. 4G).

### 3.2 Early estrogen deficiency causes an increase in vascular canal diameter compared to healthy controls

We analysed the temporal changes in vascular porosity due to estrogen deficiency by performing micro-CT analysis and BSEM on the distal femur of short and long term estrogen deficient animals (Fig. 4). At micro-CT resolutions there was no significant difference in vascular diameter, vascular porosity or vascular density in OVX animals compared to SHAM animals for either the short or longer term time points (Fig. 4A, B, C).

By BSEM, cortical bone was observed to be more porous in short term estrogen deficiency (week 4 OVX) (Fig. 4D) and a significant increase in vascular canal diameter was seen, when compared to healthy controls (week 4 SHAM) (*p* < 0.0001) (Fig. 4E). In week 14 animals, there was a decrease in vascular canal diameter in both SHAM and OVX animals compared to week 4 counterparts (p < 0.0001) (Fig. 4E). An increase in the percentage area occupied by vascular canals in week 4 OVX animals when compared to week 4 SHAM animals was also observed (*p* < 0.05) (Fig. 4F). No significant difference was observed in the percentage area occupied by vascular canals in week 14 animals compared to week 4 animals for either SHAM or OVX groups (Fig. 4F).

### 3.3 Temporal changes in lacunar microporosity and density following estrogen deficiency

**Fig. 5:**
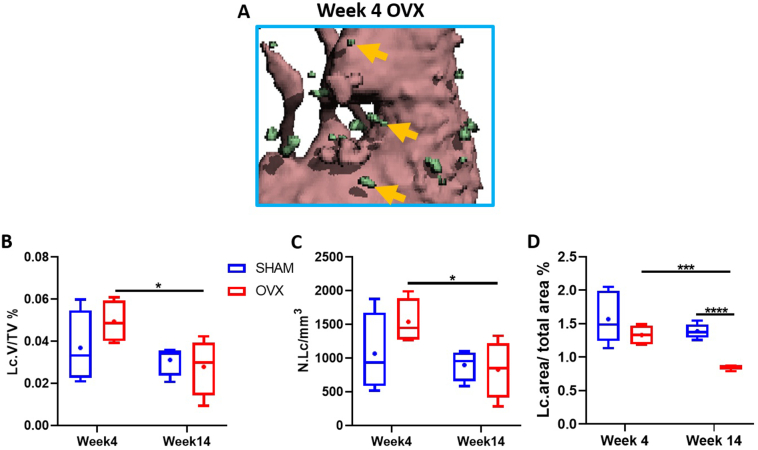
Micro-CT lacunar microporosity measurements for cortical bone from the medial region of the distal femur metaphysis for OVX groups after short (week 4) and long term (week 14) estrogen deficiency and SHAM counterparts. (A) Representative 3D reconstruction of week 4 OVX animal showing segmented osteocyte lacunae (green), (B) Lacunar porosity, Lc.V/TV (%), (C) Lacunar density, N.LC/mm^3^, (D) BSEM analysis of percentage area taken up by lacunar in a given area (%). Lc = lacunar, TV = total volume, N = number. n = 4 per group. * = *p* < 0.05.

We analysed the temporal changes in lacunar-canalicular porosity due to estrogen deficiency by performing thresholding to segment and quantify osteocyte lacunae from micro-CT analysis (Fig. 5A) and through quantitative analysis of osteocyte lacunae in the BSEM image data set (Fig. 4D).

At micro-CT resolutions there was no significant change in lacunar porosity or density for control animals between week 4 and 14 (*p* < 0.05) (Fig. 5B, C). However, in long term estrogen deficiency (week 14 OVX) there was a significant decrease in both lacunar porosity and density, when compared to OVX week 4 animals (p < 0.05) (Fig. 5B, C). There was no significant difference in lacunar porosity and lacunar density for either the short or long term estrogen deficiency groups when compared to the SHAM animals at micro-CT resolutions (Fig. 5B, C). BSEM analysis revealed that percentage area occupied by lacunae significantly decreased in week 14 OVX animals when compared to week 4 OVX animals (*p* < 0.001) (Fig. 5D).

### 3.4 Temporal changes in osteocyte lacunar and canalicular diameter following estrogen deficiency

**Fig. 6:**
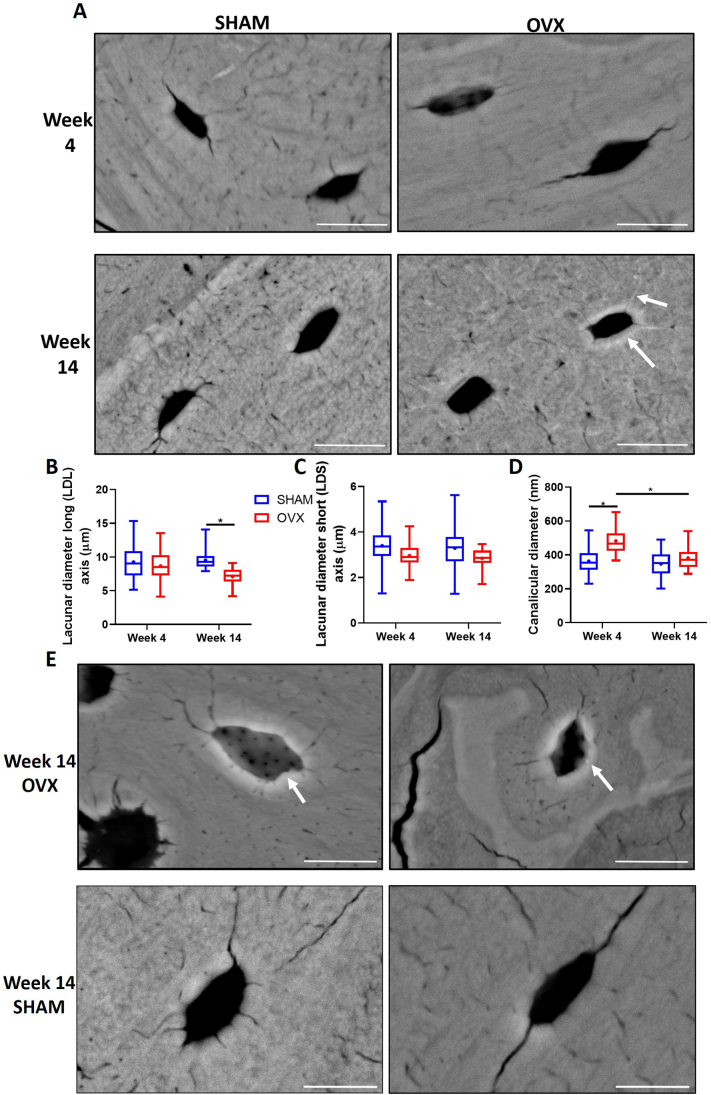
Backscatter electron imaging quantitative analysis. (A) Representative images of lacunae from week 4 and week 14 SHAM and OVX animals, (B) Lacunar diameter at the long axis (μm), (C) Lacunar diameter at the short axis (μm), (D) Canalicular diameter (nm), (E) Additional BSEM representative images from week 14 OVX and SHAM animals, demonstrating that OVX animals had more lacunae with a mineralised border around the lacunar space (white arrows). n = 4 animals per group, ≥15 lacunae per specimen randomly selected in a region of the medial cortical bone (similar to micro-CT). Scale bar = 10 μm. * = p < 0.05.

Backscatter electron imaging (Fig. 6A) was performed to analyse changes in lacunar and canalicular diameter in both short and long term estrogen deficiency, which might indicate whether mineral infilling or perilacunar resorption occurred. There was no significant difference in lacunar diameter (long and short axis) between SHAM and OVX animals at week 4 (Fig. 6B). A significant decrease in lacunar diameter (long axis) in week 14 OVX animals compared to week 14 SHAM animals was observed (p < 0.05) (Fig. 6B), but there were no significant differences in lacunar diameter (short axis) (Fig. 6C). Moreover, while osteocytes in healthy mature bone were elliptical (Long Axis Diameter: 9.53 ± 1.27, Short Axis Diameter: 3.29 ± 0.73), in bone tissue from week 14 OVX animals a decrease in the long-axis lacunar diameter without associated changes in the short axis provides evidence that the osteocytes were more circular (Long Axis Diameter: 7.09 ± 1.29, Short Axis Diameter: 2.84 ± 0.41), see Fig. 6(B, C). Qualitative analysis confirmed the observation of circular osteocytes in estrogen deficiency, and more lacunae exhibited a mineralised border around the lacunar space compared to week 14 SHAM animals (Fig. 6E).

Canalicular measurements from the BSEM images revealed an increase in canalicular diameter in week 4 OVX animals compared to week 4 SHAM animals (p < 0.05). However, there was a significant decrease in canalicular diameter in OVX animals at week 14 compared to those analysed at week 4 OVX (p < 0.05) (Fig. 6D).

### 3.5 Long-term estrogen deficiency results in an increase in unoccupied lacunae and increased incidence of MMP14+ osteocytes

**Fig. 7:**
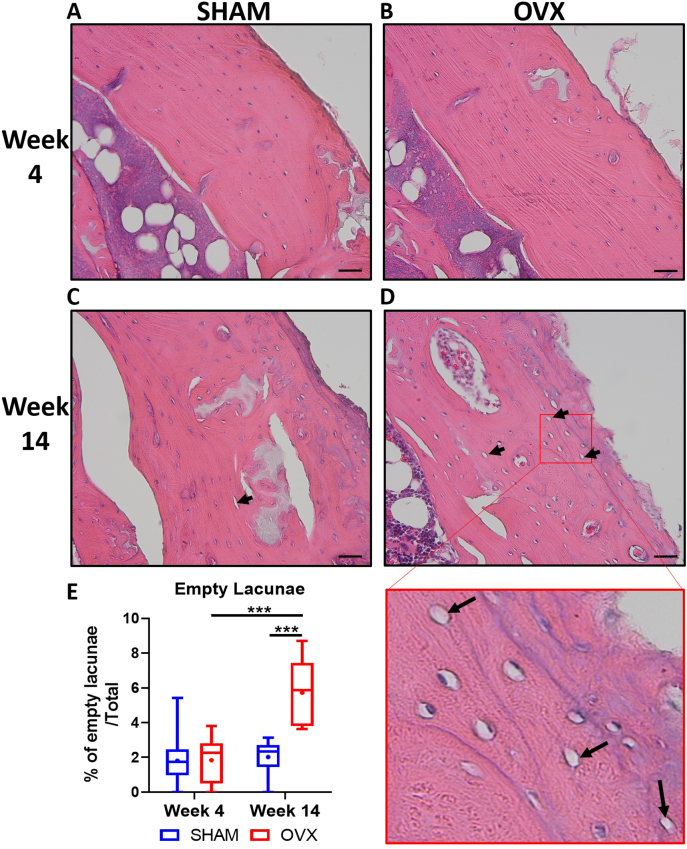
Long-term estrogen deficiency (week 14 OVX) results in an increase in unoccupied lacunae. Representative image showing Haematoxylin and Eosin (H&E) staining of cortical bone from (A) week 4 SHAM animals, (B) week 4 ovariectomized (OVX) animals, (C) week 14 SHAM animals (D) week 14 OVX animals (E) ImageJ analysis was performed to quantify the percentage of empty lacunae in a given area. Black arrows indicate empty lacunae. n = 4 per group. Scale bar = 20 μm. *** = p < 0.001.

After establishing that estrogen deficiency affected lacunar parameters in a temporal manner, the effects of estrogen deficiency on lacunar occupancy was then assessed. Haematoxylin and Eosin (H&E) staining revealed no significant differences in lacunar occupancy in week 4 SHAM compared to week 4 OVX animals (Fig. 7A, B). However, a significant increase in the amount of empty lacunae was observed in long term estrogen deficiency (week 14 OVX) compared to week 14 SHAM animals (*p* < 0.001) (Fig. 7C, D, E). There also was a significantly higher incidence of empty lacunae in week 14 OVX compared to short term estrogen deficient animals (week 4 OVX) (p < 0.001) (Fig. 7B, C, E).

**Fig. 8:**
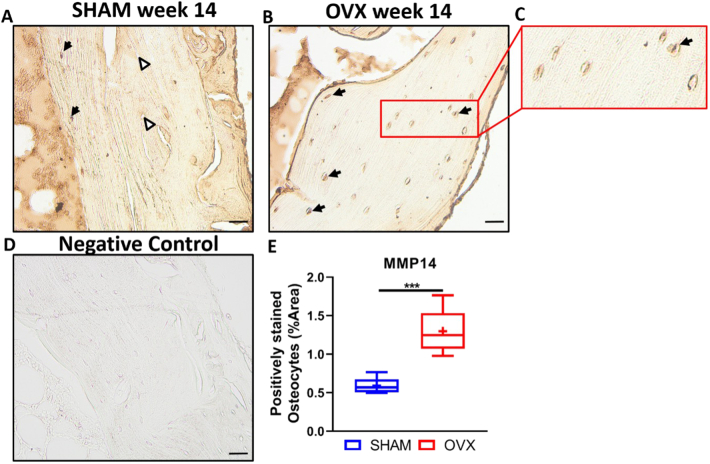
Week 14 OVX animals display an increase in MMP14+ osteocytes compared to week 14 SHAM animals. Representative images showing representative MMP14+ staining in (A) week 14 SHAM rat, (B) week 14 ovariectomized (OVX) rat (C) magnified image (red box) of OVX week 14 rat, (D) negative control-antibody diluent instead of primary antibody was used with the secondary antibody, (E) ImageJ analysis was performed to assess the prevalence of positively stained osteocytes, normalised to total bone area. Black arrows = MMP14+ osteocytes, white triangle = osteocyte that is not positive for MMP14. n = 4 per group. Scale bar = 20 μm. *** = p < 0.001.

Since most of the changes in lacunar parameters occurred during long term estrogen deficiency (week 14 OVX) and a reduction in mean mineral density was also observed, week 14 cortical bone was stained for MMP14, a matrix metalloprotease involved in perilacunar resorption. A significant increase in MMP14+ osteocytes was observed in tissue from week 14 OVX animals compared to controls (week 14 SHAM) (p < 0.001) (Fig. 8A, B, C, E).

## 4 Discussion

In this study, temporal changes in lacunar and vascular porosity in estrogen deficiency are reported. In short term estrogen deficiency (4 weeks post-OVX) increases in vascular diameter arise, whereas in long term estrogen deficiency (14 weeks post-OVX) a decrease in lacunar porosity and lacunar diameter were reported. These longer term changes coincided with an increased prevalence of empty lacunae and the observation of more circular osteocyte lacunae with mineralised borders, which might provide an explanation for the decrease in lacunar porosity and lacunar diameter. In addition there was an increase in the prevalence of MMP14+ osteocytes. Together these results provide an insight into the temporal changes that occur in cortical microporosity during estrogen deficiency and suggest the likelihood of occurrence of perilacunar resorption and micropetrosis.

There are a number of limitations to this study, which need to be considered. Firstly, the estrogen deficient rat model may differ from human osteoporosis since there are biological differences between rats and humans (Lelovas et al., 2008). However, the ovariectomized rat is a well-established model, due to similarities with respect to bone loss and calcium absorption (Kalu, 1991; Rosen, 2000), which are a characteristic of human postmenopausal osteoporosis and also skeletal responses to drug treatments and exercise (Lin et al., 1994; Li et al., 2009; Recker et al., 2015; Sanudo et al., 2017). Secondly, the highest isotropic resolution achievable on the micro-CT system (μCT 100) used was 2 μm and our approach recognised lacunae as pores with volume of at least 100 μm^3^, following a previous approach that demonstrated to be effective in assessing vascular microarchitecture (Palacio-Mancheno et al., 2014). However, this micro-CT resolution may have underestimated the lacunar density and porosity, and others have also used 1 μm resolution for such studies (Palacio-Mancheno et al., 2014). Nonetheless, the lacunae not detected by a resolution of 2 μm would be excluded from both groups in this study and therefore the comparison between SHAM and OVX is valid. Moreover, as we can detect significant differences at the resolution limitations of micro-CT and BSEM, we expect the differences to be more pronounced using higher resolution approaches. Another limitation of our approach might be that two layers of surface voxels from segmented surfaces (i.e. endosteal, periosteal, vascular pores and lacunae surfaces in cortical bone and from each trabecula) were excluded from BMDD analysis, to reduce partial volume effects. This would remove 4 μm of the segmented surface (2 × 2 μm voxels), meaning that subtle changes in mineral around lacunae, potentially due to micropetrosis or perilacunar resorption, may not be detected by micro-CT. Nonetheless, despite the removal of these surface voxels, alterations in cortical mineralisation were detected in long-term estrogen deficient animals. Moreover, micro-CT was performed at such a high resolution to detect pores (vascular, lacunar) that sphere fitting to determine cortical bone thickness could not be performed. Our backscatter electron imaging analysis enabled cortical thickness measurements and a higher resolution study of both microporosity and mineralisation, but this 2D approach does not take into consideration the orientation of the ellipsoidal osteocytes within the bone. Thus, our combined approach may not capture all the changes that occur in the lacunar-canalicular network and high resolution synchrotron micro-CT scanning or serial section SEM or TEM imaging should be considered to further investigate the results presented in this study. Finally, the animal sample sizes were determined based on a-priori power calculations to detect significant changes in bone loss, but might not suffice to detect subtler changes in microporosity. Although analysis of the microporosity data in this pilot study (post hoc) indicated that these sample sizes may provide sufficient power with respect to lacunar-canalicular diameter measurements at 14 weeks (>~80%), future studies with larger sample sizes are required to explore further the interesting findings of this pilot study.

Similar to a previous in vivo micro-CT study from our group (O'Sullivan et al., 2020), we reported a significant reduction in bone volume fraction and trabecular number with no change in trabecular thickness or tissue mineralisation in the ovariectomized rat tibiae in the first four weeks of estrogen deficiency. Interestingly, recent in vitro studies have demonstrated the role of mechanically stimulated osteocytes in pro-osteoclastogenic paracrine signalling (RANK/OPG) (Geoghegan et al., 2019; Allison and McNamara, 2019), osteoclastogenesis and resorption (Allison and McNamara, 2019; Allison et al., 2020). Moreover, we also report a significant increase in trabecular thickness in the distal femurs from long term estrogen deficient animals, which is in keeping with changes reported for the tibia in (O'Sullivan et al., 2020). Yet, unlike the tibia analysis of (O'Sullivan et al., 2020), we did not detect mineralisation changes in the trabecular bone from the distal femurs of OVX animals. This supports previous qBEI studies, which reported no difference in mean mineral density between OVX and aged matched controls in the vertebrae and femur at 12 weeks, 48 weeks (Kneissel et al., 2001) and 20.5 months (Valenta et al., 2005). In addition, ex-vivo SRμCT studies have also reported no difference in mean mineral density in the lumbar vertebrae and tibia of OVX rats compared to age matched controls (Campbell et al., 2011). The secondary mineralisation changes observed in the tibia in our previous in vivo study (O'Sullivan et al., 2020), might be region-specific or may occur at a later stage in the femur than the 14 week time-point studied herewith. However, further studies analysing different regions and later time points would need to be performed to determine this.

Lacunar porosity obtained from micro-CT analysis herein was approximately 0.04% in femurs from early-stage SHAM rats, which is a lower than values reported previously for rat proximal tibiae (1.5% using 1 μm resolution) (Sharma et al., 2018; Palacio-Mancheno et al., 2014), mice mid-diaphysis femur (1.3% using 0.7 μm resolution) (Schneider et al., 2007) and rat mid-diaphysis femur (1.5% using 0.75 μm resolution) (Tommasini et al., 2012). This difference is likely due to the isotropic resolution of 2 μm not being adequate to capture all lacunae. However, when high resolution backscatter electron imaging was performed it was found that lacunar porosity (lacunae percentage area/total) for short term SHAM animals to be 1.57 ± 0.37%, which is similar to that previously reported in rodents (Sharma et al., 2012; Sharma et al., 2018; Schneider et al., 2007; Tommasini et al., 2012).

It has previously been reported that lacunar porosity increased in ovariectomized rats early in the disease (6 week post ovariectomy) when compared to SHAM animals and this was proposed to arise due to an increase in canalicular size and not osteocyte lacunar density, or lacunar size (Sharma et al., 2012; Sharma et al., 2018). Similarly, we report an early increase in canalicular diameter at 4 weeks post ovariectomy but no changes in lacunar density and size were observed in short term estrogen deficiency. We report for the first time that canalicular diameter was in fact reduced in longer term estrogen deficiency (14 weeks). However, a decrease in lacunar porosity and density arose in long term estrogen deficiency, which was accompanied by a decrease in lacunar diameter (long axis). Interestingly, this finding is comparable to other studies that reported a reduction in lacunar area (Mullender et al., 1996) and lacunar density (Mullender et al., 2005; Qiu et al., 2003) in female osteoporotic patients.

The decrease in canalicular and lacunar diameter may be due to the beginning of micropetrosis (Frost, 1960), and our backscatter electron imaging provided evidence of a higher incidence of mineralisation around the edge of the lacunae, although no fully mineralised lacunae were identified for the duration of this study. Nonetheless, histologically, a reduction in lacunar occupancy was also observed, which has been shown to be one of the first events that occurs before hyper mineralisation and mineral infilling (Frost, 1960). Moreover, osteocyte apoptosis has also been reported to increase in osteoporotic bone (Tomkinson et al., 1998; Almeida et al., 2007; van Essen et al., 2007; Emerton et al., 2010). The reduction in lacunar diameter (long axis) resulted in more circular lacunae, and it would therefore be expected that any osteocytes resided in these lacunae may also be more rounded. Interestingly, an in vitro study revealed that round osteocytes were more mechanosensitive (Bacabac et al., 2008). In addition, the changes in lacunar shape and canalicular diameter may also impact the transfer of mechanical signals to the cells, through tethering elements and integrins (Wang et al., 2007; McNamara et al., 2009). Thus one possible explanation for the reduction in lacunar diameter to produce a more circular lacunar may be a mechanobiological response to restore the mechanosensitivity of the osteocytes, which has been shown to be impaired during estrogen deficiency (Deepak et al., 2017; Geoghegan et al., 2019).

An increase in MMP14+ osteocytes was seen in ovariectomized rat cortical bone, which also might suggest an increase in perilacunar resorption in the long term estrogen deficient animals compared to long term SHAM animals. However, it was found that lacunar diameter was reduced in these OVX animals. Thus, it may be possible that a subset of osteocytes are undergoing apoptosis leading to micropetrosis and mineral infilling, while the remaining osteocytes are resorbing their surrounding matrix. Further analysis should be performed such as a microarray on the cortical tissue looking at expression of genes involved in perilacunar resorption, e.g. CTSK, carbonic anhydrase, MMP13, as well as analysis of intracellular osteocyte acidification (Yee et al., 2019), in order to provide a more detailed understanding of the microporosity and perilacunar changes during estrogen deficiency.

An increase in vascular diameter and vascular porosity was observed in short term estrogen deficient animals, which supports previous studies which reported an increase in vascular porosity and diameter in week 6 post-ovariectomy animals compared to SHAM animals (Sharma et al., 2018). Vascular porosity in short term SHAM animals was 1.78 ± 0.52% as determined by micro-CT analysis, which falls in the range of values reported in literature for rats (1.7 ± 0.6–2.76 ± 1.11%) using micro-CT, synchrotron based micro and Nano-CT as well as histological analysis (Matsumoto et al., 2006; Schneider et al., 2007; Britz et al., 2010). The increase in vascular porosity and diameter, seen in short term estrogen deficiency has been shown through finite element analysis to have a negative impact on interstitial fluid flow through the lacunar-canalicular network in cortical bone (Gatti et al., 2018).

The decrease in canalicular and lacunar diameter would both contribute to altered fluid velocity through the lacunar canalicular system and may affect transport of nutrients between osteocytes and the blood vessels, which can lead to osteocyte apoptosis and might induce up-regulation of osteoclastogenic factors and increase bone resorption (Cardoso et al., 2009; Kennedy et al., 2014).

Computational modelling has been applied to investigate how lacunar-canalicular changes alter mechanical stimulation of osteocytes (Gatti et al., 2018; Verbruggen et al., 2016; Verbruggen et al., 2015). A study revealed that load-induced interstitial fluid flow around osteocyte lacunae was enhanced in bone from OVX rats (Ciani et al., 2014). Previous computational work from our group revealed that osteocytes that were in close proximity to a Volkmann canal experience strains that exceed the strain stimulus for osteogenesis (>10,000 με) due to both strain amplification and relieving effects of the vascular cavity (Vaughan et al., 2013). Additionally, another study from our group found that in short term estrogen deficiency (week 5 post-OVX), a greater proportion of the osteocyte (10%) experienced osteogenic strains (>10,000 με) than those in control SHAM animals (Verbruggen et al., 2015). This could at least be in part due to osteocytes being in closer in proximity to vascular canals due to increases in vascular porosity in short term estrogen deficiency. In long term estrogen deficiency, the vascular canal density as well as vascular diameter decreased compared to week 4 OVX animals. This along with the increase in trabecular thickness also observed in this study suggests that bone may have adapted to alter the mechanical environment, perhaps in an attempt to restore homeostasis. In keeping with this, our group previously reported that in long term estrogen deficient rats (34 weeks post-ovariectomy), cellular strains experienced by osteocytes decreased significantly compared to the strain experienced in short term estrogen deficiency (5 weeks post-ovariectomy) to levels comparable to control SHAM animals (Verbruggen et al., 2015). Increases in microporosity have been shown to decrease bone mineral density, bone stiffness and most importantly bone strength (Currey and Shahar, 2013; Osterhoff et al., 2016). In addition to changes in lacunar-canalicular porosity, a reduction in cortical thickness was observed, as well as a decrease in mean and mode mineral density in long term estrogen deficient rats, which is similar to what has been reported for human osteopenic and osteoporotic patients (Melton et al., 2007; Richards et al., 2010).

## 5 Conclusion

This pilot study provides evidence of temporal changes in cortical microporosity arising during estrogen deficiency in a rat model of osteoporosis. We report that lacunar and vascular porosity increase in short term estrogen deficiency, which coincides with the period in which rapid bone loss occurs, whereas in long-term estrogen deficiency lacunar and canalicular diameter were shown to decrease. These changes coincided with an increased prevalence of empty lacunae and the observation of more circular osteocytes with a mineralised border around the lacunar space. In addition we report an increase in MMP14+ osteocytes, which also suggests active matrix degradation by these cells. On this basis we propose that microporosity changes arise due to processes driven by distinct populations of osteocytes, which are either actively resorbing their matrix or have undergone apoptosis and are infilling lacunae by micropetrosis.

## CRediT authorship contribution statement

**H. Allison:** Conceptualization, Methodology, Investigation, Formal analysis, Data curation, Visualization, Software, Validation, Writing – original draft, Writing – review & editing. **L.M. O'Sullivan:** Methodology, Investigation, Formal analysis, Data curation, Software, Validation. **L.M. McNamara:** Conceptualization, Supervision, Funding acquisition, Project administration, Resources, Formal analysis, Writing – original draft, Writing – review & editing.

## Declaration of competing interest

The authors declare that they have no known competing financial interests or personal relationships that could have appeared to influence the work reported in this paper.
